# Connecting Metabolic Rewiring With Phenotype Switching in Melanoma

**DOI:** 10.3389/fcell.2022.930250

**Published:** 2022-07-15

**Authors:** Paola Falletta, Colin R. Goding, Yurena Vivas-García

**Affiliations:** ^1^ Vita-Salute San Raffaele University, Milan, Italy; ^2^ Experimental Imaging Center, IRCCS Ospedale San Raffaele, Milan, Italy; ^3^ Nuffield Department of Clinical Medicine, Ludwig Cancer Research, University of Oxford, Oxford, United Kingdom

**Keywords:** metabolic plasticity, melanoma, MITF, fatty acids, mitochondria, heterogeneity, drug resisitance

## Abstract

Melanoma is a complex and aggressive cancer type that contains different cell subpopulations displaying distinct phenotypes within the same tumor. Metabolic reprogramming, a hallmark of cell transformation, is essential for melanoma cells to adopt different phenotypic states necessary for adaptation to changes arising from a dynamic milieu and oncogenic mutations. Increasing evidence demonstrates how melanoma cells can exhibit distinct metabolic profiles depending on their specific phenotype, allowing adaptation to hostile microenvironmental conditions, such as hypoxia or nutrient depletion. For instance, increased glucose consumption and lipid anabolism are associated with proliferation, while a dependency on exogenous fatty acids and an oxidative state are linked to invasion and metastatic dissemination. How these different metabolic dependencies are integrated with specific cell phenotypes is poorly understood and little is known about metabolic changes underpinning melanoma metastasis. Recent evidence suggests that metabolic rewiring engaging transitions to invasion and metastatic progression may be dependent on several factors, such as specific oncogenic programs or lineage-restricted mechanisms controlling cell metabolism, intra-tumor microenvironmental cues and anatomical location of metastasis. In this review we highlight how the main molecular events supporting melanoma metabolic rewiring and phenotype-switching are parallel and interconnected events that dictate tumor progression and metastatic dissemination through interplay with the tumor microenvironment.

## Introduction

Genetic and non-genetic intra-tumor heterogeneity define the coexistence of phenotypically and metabolically different cancer cell subpopulations within the same tumor and are one of the major features of tumor complexity (Heppner, 1984). It is increasingly recognized that non-genetic intra-tumor heterogeneity (from here simply defined as heterogeneity) is one of the major barriers for developing successful therapies and a cause of cancer relapse (Marusyk et al., 2020), and has a remarkable impact on metastasis formation (Lawson et al., 2018).

Metastasis, the major cause of cancer-related death, is a very inefficient process. For a metastatic lesion to occur, cancer cells undergo a series of phenotypic changes that allow the acquisition of migratory capacity, the ability to survive in circulation and at the destination tissue, and the potential to proliferate when the seeding organ has been reached. Although millions of cancer cells are able to escape from the primary tumor into the blood or lymph, very few succeed at initiating a secondary lesion in a distant organ (Lu et al., 2015).

To escape from and survive outside of the primary tumor, invasive tumor cells require metabolic flexibility, first to fuel the high energy demanding migratory process (Cunniff et al., 2016; Zanotelli et al., 2018), and second to endure the nutrient-limiting environments that they might encounter. Therefore, tumor cells rely on metabolic rewiring, which allows prompt and reversible adaptation to the energetic and biosynthetic requirements of each step of metastatic dissemination. Moreover, the different and dynamic microenvironments encountered by a migrating tumor cell could be themselves drivers of metabolic changes (Joyce and Pollard, 2009; Boroughs and Deberardinis, 2015). Thus, the main drivers of metastasis are likely to be reversible changes within and external to the primary tumor which lead to transcriptional, translational, and metabolic rewiring that trigger metastatic dissemination.

Melanoma, the deadliest form of skin cancer, arises from the oncogenic transformation of cells belonging to the melanocytic lineage (Shain and Bastian, 2016) and represents a paradigm for understanding phenotypic heterogeneity and the mechanisms underpinning metastatic dissemination since there are excellent markers that highlight specific phenotypic subpopulations. Moreover, remarkable progress has been made in understanding the metabolism of specific melanoma subpopulations and the impact of microenvironmental factors such as hypoxia (Feige et al., 2011; Cheli et al., 2012; Widmer et al., 2013; Louphrasitthiphol et al., 2019), nutrient limitation (Falletta et al., 2017; Ferguson et al., 2017), inflammatory signals or exposure to chemotherapy (Landsberg et al., 2012; Riesenberg et al., 2015; García-Jiménez and Goding, 2019; Rambow et al., 2019) on phenotypic identity and melanoma gene expression programs.

Considering that cell metabolism is tightly associated with both phenotypic identity and the adaptation to local and systemic microenvironmental factors, it is not surprising that changes in the tumor microenvironment (TME) have an impact on tumor metabolism and melanoma cell fate. Furthermore, recent evidence suggests that other factors such as anatomical location, which might itself be subjected to specific microenvironmental cues, have an impact on melanoma cell features (Weiss et al., 2022).

In this review we focus on the metabolic traits that characterize the different phenotypic states of melanoma cells and the metabolic reprogramming that might dictate the transitions between states that drive melanoma progression and metastasis. Since the role of glucose and glutamine metabolism in melanoma has been widely discussed in recent well-documented reviews (Ratnikov et al., 2017; Avagliano et al., 2020), here we will focus on fatty acid metabolism. In particular, we will describe the molecular drivers of metabolic plasticity and focus on the relevance of lipid usage as an alternative nutrient to glucose and glutamine during the metastatic process. Moreover, we highlight the interconnections between melanoma metabolism and the TME, and how a better understanding of the molecular and metabolic basis of melanoma heterogeneity may facilitate the identification of possible targets for therapeutic intervention.

## Interplay Between Phenotypic Heterogeneity and Metabolic Flexibility in Melanoma

Melanoma is characterized by high levels of genetic heterogeneity and displays one of the highest somatic mutational burdens among all cancers (Pleasance et al., 2010; Reddy et al., 2017). Some of the most frequent and well-characterized mutations found in cutaneous melanoma affect the proto-oncogenes BRAF, accounting for more than 50% of cases, and NRAS, found in 10%–25% of patients (Flaherty et al., 2012; Akbani et al., 2015). Moreover, almost 20% of cases show mutations leading to PTEN loss-of-function (Pollock et al., 2002; Goel et al., 2006). These genetic alterations induce activation of the MAPK and PI3K/AKT pathways, both well-known regulators of cell metabolism, affecting processes such as cell growth, proliferation, and survival (Meier et al., 2005; Flaherty et al., 2012). Interestingly, differential and exclusive genetic alterations between cutaneous and acral melanomas have been recently reported to control these pathways (Weiss et al., 2022). Thus, acral melanomas show enrichment in Crk-like protein (CRKL) amplification, that in cooperation with transcriptional programs expressed in specific anatomical locations, such as the limbs, lead to the amplification of insulin-like growth factor (IGF) signaling by interfering with the PI3K pathway downstream the IGF signaling itself. In addition, this finding suggests the existence of differential oncogenic susceptibilities depending on melanoma location. However, whether such a disparity is affected by microenvironmental factors remains unclear.

Although the study of these genetic alterations is essential for the understanding of the mechanisms involved in melanoma initiation, evidence suggests that the melanoma mutational landscape is not sufficient to explain why phenotypic heterogeneity and metastatic dissemination occur. Although oncogenic mutations might facilitate the metastatic process (Jamal-Hanjani et al., 2015; Pogrebniak and Curtis, 2018), they are unlikely to be the main drivers, since the time necessary for new pro-metastatic mutations to arise is longer than the period in which metastases normally emerge (Jones et al., 2008). A recent study performed across 50 different tumor types revealed that some mutations can be frequently found in tumors with specific metastatic patterns. In melanoma, for example, NF1 mutations are prevalent in lung metastasis while a higher frequency of PTEN mutations is found in brain metastasis. However, the authors revealed the absence of unique mutations or set of mutations that could serve as reliable indicators to predict metastatic behavior (Nguyen et al., 2022). Moreover, primary tumors and metastases very often share the same mutational burden (Makohon-Moore et al., 2017), supporting the role of mutations in metastases initiation, but not as the governors of the metastatic process (Vanharanta and Massagué, 2013; Patel et al., 2021).

Genetic intra-tumor heterogeneity may impose tumor metabolic heterogeneity. Indeed, most human tumors present somatic mutations, many of which are likely to affect metabolism (Martincorena et al., 2015). A compelling example in melanoma are BRAF mutations which confer increased glycolytic capacity compared to BRAF wild type tumors (Shi et al., 2017). However, oncogenic BRAF seems not to be sufficient to generate specific metabolic signatures that allow metastatic and non-metastatic melanomas to be differentiated (Shi et al., 2017). This means that beyond the role of specific mutations or individual pathways, cancer metabolic rewiring dictating melanoma fate may rely on the concerted action of more than one driver. Thus, while a specific genetic mutation may provide a cell with an adaptive and pro-survival advantage to face a specific metabolic or nutritional stress, the same genetic feature may create a selective vulnerability when the same cell is subjected to a different insult (McGranahan and Swanton, 2015). Indeed, the major characteristics of metabolic rewiring are flexibility and reversibility, which confer survival advantages upon many different metabolic insults. For this reason, genetic alterations that normally lead to non-reversible phenotypes, unless a new mutation arises, may not be sufficient to explain either tumor phenotypic heterogeneity or metabolic flexibility as aspects that promote metastatic dissemination (Pogrebniak and Curtis, 2018; Kim and DeBerardinis, 2019).

Melanoma phenotypic heterogeneity is characterized by specific transcriptional programs, activation of which depends on the interplay between genetic and microenvironmental inputs. More than a decade ago, the seminal work of Hoek et al. (2006) identified by hierarchical clustering two distinct phenotypes, namely the proliferative and the invasive cohorts, which reflect differences in metastatic potential. The proliferative cohort expresses genes critical to neural crest differentiation and cell cycle control, while the invasive cohort is enriched with genes involved in invasive and metastatic behavior. Strikingly, the phenotypic traits associated with these two cohorts showed no correlation with the genetic mutational landscape but could be associated instead with specific transcriptional programs. Indeed, the two cohorts segregate the expression of the MIcrophthalmia-associated Transcription Factor (MITF), a master regulator of the melanocyte lineage (Goding and Arnheiter, 2019), and its downstream targets. Specifically, in the proliferative cohort MITF gene expression is upregulated, while in the invasive cohort low levels of MITF are detected, suggesting a direct involvement of MITF in cell fate regulation. Two years later the same group demonstrated that *in vivo* melanoma cells may switch between these states, and since tumors show evidence for both proliferative and invasive cell types it is likely that microenvironmental conditions drive this transcriptional switching and determine cell plasticity (Hoek et al., 2008). MITF-positive cells can be found at the site of metastasis, confirming the ability of melanoma cells to switch to and from these transcriptional states (Beleaua et al., 2021). Moreover, distinct transcriptional states characterized by variable MITF expression and activity show different responses to MAPK pathway inhibitors, highlighting how tumor heterogeneity and plasticity could be drivers of MAPK inhibitor resistance (Roesch, 2015). Indeed, pharmacologically forcing the expression of MITF drives melanoma cells towards a differentiated cell state, which can be exploited therapeutically (Sáez-Ayala et al., 2013). Collectively these observations fulfill the prediction, based on experiments where MITF levels were experimentally manipulated, that microenvironments that decrease MITF expression would trigger invasion while those that increase MITF would promote proliferation or differentiation (Carreira et al., 2005, Carreira et al., 2006).

More recently, this notion has been corroborated and widened by a series of studies: The proliferative and invasive cell states have been recapitulated by RNA-sequencing analysis of tumor biopsies by Verfaillie et al. (2015), which further highlighted both the role of chromatin remodeling in transcription reprogramming dictating phenotypic plasticity and the absence of correlation with any specific mutation in known melanoma driver genes such as BRAF (Verfaillie et al., 2015). Single-cell RNA-sequencing (RNA-seq) of BRAF-mutant melanoma patient-derived xenografts (PDX) exposed to MAPK inhibitors revealed up to four distinct subpopulations of drug-tolerant cells co-occurring within the same post-therapy minimal residual disease (MRD) and established through non-mutational adaptive events (Rambow et al., 2018). These states are characterized by high (the pigmented state), intermediate (the starved state), and low (the invasive and neural crest stem cell states) levels of MITF expression and activity. Furthermore, Tsoi et al. (2018) revealed that melanoma differentiation involves four progressive subtypes which fall in a two-dimensional differentiation trajectory by performing a gene expression comparative analysis of a panel of human melanoma cell lines with an *in vitro* model of melanocyte differentiation. The states identified by Tsoi et al. (2018), largely correspond to those identified by Rambow et al. (2018) and Rambow et al. (2019). Indeed, the phenotypic plasticity of melanoma may be attributed to the embryonic origins of melanocytes, which derive from the multi-potent and highly migratory neural crest population (Sauka-Spengler and Bronner-Fraser, 2008).

Strikingly, different phenotypic states exhibit distinct metabolic profiles. For example, increased lipid anabolism is associated with proliferation (Currie et al., 2013; Peck et al., 2016), while increased dependence on exogenous fatty acids (FA) is linked to invasion and metastatic dissemination (Pascual et al., 2017; Zhang et al., 2018). Therefore, metabolic flexibility itself appears as a hallmark of heterogeneity, and consequently it is likely to be driven by the concerted action of specific oncogenic programs or acquired mutations and external cues imposed by a dynamic microenvironment (Kim and DeBerardinis, 2019).

In this still evolving scenario, a critical concept has emerged: while driver mutations provide a selective growth advantage to melanoma cells and promote cancer progression, the TME can reprogram the transcriptional landscape directing cells towards different phenotypic states that may coexist within the same tumor (Quail and Joyce, 2013). Furthermore, oncogenic mutations may have the crucial role of lowering the threshold above which a melanoma cell can transition between different phenotypic states, for example by sensitizing cells to microenvironmental stress and leading to the acquisition of the invasive phenotype (Bernards and Weinberg, 2002; Hoek and Goding, 2010; Ganesh and Massagué, 2021).

The identification of the events dictating phenotypic heterogeneity and plasticity is therefore a pivotal aspect, which may allow the development of new strategies to target melanoma progression and metastatic dissemination.

## The Role of MITF in Metabolic Flexibility and Cell Fate Determination

The current knowledge about melanoma metabolism reveals a growing complexity that involves the coordinated participation of different pathways and metabolic activities in the acquisition of specific phenotypes. This flexibility allows the cells within the tumor to switch from one preferential metabolic substrate to another, depending on the restrictions imposed by the TME or by specific oncogenic programs. Thus, metabolic rewiring is such an important process that melanoma cells usually show enhanced activity of general metabolic effectors such as BRAF and NRAS, which activate the downstream MAPK pathway, or the loss of PTEN and activation of the PI3K/AKT pathway, to increase their metabolic capacity. Moreover, melanoma cells display high activity of the transcription factor MYC (Kraehn et al., 2001), a master regulator of cell metabolism that operates as a well-known oncogene in melanoma and other cancer types (Stine et al., 2015; Ratnikov et al., 2017).

**FIGURE 1:**
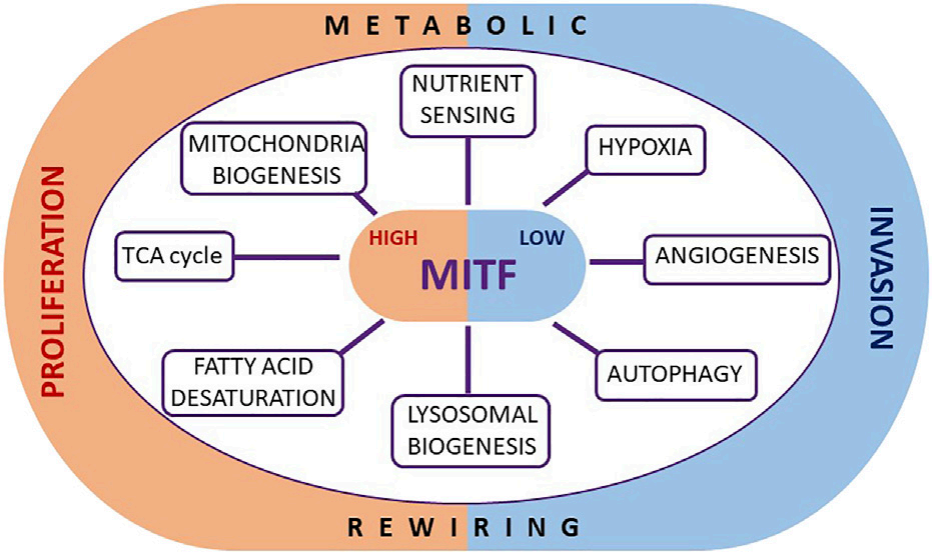
Metabolic roles of MITF in melanoma. The transcription factor MITF, known to regulate melanoma plasticity and cell identity, may play such a role also through the direct regulation of specific genes crucially involved in several metabolic functions. By modulating metabolic events such as the TCA cycle, mitochondrial biogenesis, nutrient sensing, the hypoxia response, angiogenesis, autophagy, lysosome biogenesis and fatty acid desaturation, MITF tunes cell proliferation, and prevents the stabilization of an invasive phenotype.

Beyond the implication of widely recognized metabolic molecular pathways common to all solid tumors, melanoma also possess lineage-restricted mechanisms able to coordinate metabolism and cell proliferation. Of particular relevance is the involvement of the transcription factor MITF, which can control melanoma cell fate, at least in part as a crucial and increasingly recognized regulator of metabolism (Figure 1).

Of note, MITF is a key regulator of phenotypic identity and melanoma cell fate, able to promote differentiation and proliferation (Goding and Arnheiter, 2019). Low levels of MITF are associated with invasion, enhanced tumor-initiating capacity (Carreira et al., 2006; Cheli et al., 2012), as well as with drug-resistant and slow-cycling phenotypes (Konieczkowski et al., 2014; Müller et al., 2014; Dugo et al., 2015; Rambow et al., 2018). Importantly, nutritional and metabolic microenvironmental signals transcriptionally and translationally downregulate MITF (Falletta et al., 2017; Ferguson et al., 2017; Vivas-García et al., 2020) impairing proliferation and promoting invasion, supporting a role of MITF as central coordinator of nutrient signaling and cell behavior.

The direct involvement of MITF in melanoma proliferation, a highly nutrient-demanding state, strongly suggests that it may also participate in controlling the metabolic landscape by providing both the fuel required for the increased energy consumption of a highly replicative cell, and the metabolic bricks to build new macromolecular structures, such as membrane components and DNA. Similarly, low MITF levels presumably contribute to the establishment of the metabolic profile displayed by slow-cycling cells, as well as the metabolic abnormalities generally associated with therapy-resistance, as recently reviewed by Gonçalves et al., 2021. Given the fact that MITF levels confer phenotypic identity, and that MITF expression is suppressed by environmental and metabolic cues, it is not surprising that melanoma tumors, that are highly phenotypically heterogeneous, comprise different cells expressing variable levels of MITF that dictate their tendency to proliferate or invade (Goodall et al., 2008; Pinner et al., 2009). Since high or low MITF expressing cells very likely display distinctive metabolic features in an MITF-dependent manner, different phenotypic melanoma subsets are likely to respond differently to the same microenvironmental signals.

This is the case for the response to hypoxia that triggers differential expression of gene sets in different phenotypic melanoma subsets (Louphrasitthiphol et al., 2019). Remarkably, cells expressing low levels of MITF can also show constitutive activation of a hypoxia gene expression program owing to MITF controlling the levels of succinate that can act to stabilize hypoxia induced transcription factors (HIFs) (Louphrasitthiphol et al., 2019). This arises owing to the ability of MITF to control the tricarboxylic acid (TCA) cycle by transcriptionally regulating the enzyme succinate dehydrogenase, SDHB (Louphrasitthiphol et al., 2019), that converts succinate to fumarate. Low MITF therefore leads to reduced SDHB expression and consequently elevated succinate levels.

The interplay between HIF1α, the master regulator of hypoxic response, and MITF seems to be complex and based on a reciprocal regulation. It was initially described that MITF directly induces the expression of HIF1α (Buscà et al., 2005), and it was later shown that under hypoxia the levels of MITF are repressed by a mechanism that involves BHLHE40/DEC1 (Feige et al., 2011). This negative feedback loop is likely aimed at limiting the hypoxic response in melanoma cells. Moreover, early in the response to hypoxia, melanoma cells transiently upregulate MITF in a HIF1α-dependent manner, and, interestingly, both co-regulate specific HIF1α targets such as the angiogenic factor VEGF-A and the Sodium/Glucose Cotransporter SLC5A9 (Louphrasitthiphol et al., 2019), in an attempt to mitigate and reverse the effects of hypoxia.

Similarly, the degree of FA saturation that determines the balance between the amount of intracellular long chain Saturated Fatty Acids (SFAs) and the mono-unsaturated fatty acids (MUFAs) is also dependent on MITF. The SFA:MUFA ratio, essential for proper membrane biogenesis during cell proliferation, is directly regulated by the activity of the oxygen and iron-dependent lipogenic enzyme stearoyl Co-A desaturase 1 (SCD1), which converts long chain SFAs to MUFAs (Koeberle et al., 2016). In melanoma, MITF controls FA saturation by transcriptionally upregulating the SCD1 gene, whereby SCD1 is highly expressed in proliferative cells where levels of MITF are high, while low SCD1 levels correlate with low MITF and invasive cells (Vivas-García et al., 2020). As a consequence, cells expressing low levels of MITF have a high SFA:MUFA ratio.

In addition to promoting FA synthesis, a further functional role of MITF in regulating energy metabolism to sustain melanoma proliferation has been observed after treatment with diclofenac and lumiracoxib, two commonly used therapeutic agents that decrease melanoma proliferation by impairing oxidative and glycolytic pathways through MITF downregulation (Brummer et al., 2019). Related to oxidative metabolism and cell survival, although in physiological settings, MITF also regulates the production of Reactive Oxygen Species (ROS) in the retinal pigmented epithelium. In melanoma, MITF repression sensitizes melanoma cells to ROS levels through the direct regulation of genes involved in the response to oxidative stress (Liu et al., 2009).

Moreover, MITF directly activates the transcription of the peroxisome proliferator-activated receptor gamma 1-α (PPARGC1A or PGC1α) (Haq et al., 2013; Vazquez et al., 2013), a transcriptional coactivator that drives transcription of mitochondrial proteins and detoxification enzymes, defined as a master regulator of mitochondria biogenesis (Wu et al., 1999). PGC1α also induces MITF transcription in melanocytes and in melanoma (Shoag et al., 2013), giving rise to the possibility of a positive feedback loop. Moreover, MITF has also been reported to activate expression of NRF2, a key regulator of antioxidant signaling (Han et al., 2020), which in turns can inhibit MITF activity (Shin et al., 2014; Jessen et al., 2020), suggesting a negative feedback loop between MITF and antioxidant genes. Collectively, these observations suggest the existence of complex regulatory crosstalk between MITF and redox-related genes. Of note, loss of the tumor suppressor FBXW7 enhances mitochondria oxidative metabolism by reactivating the expression of MITF and, in turn, PGC1α (Abbate et al., 2018).

Another direct transcriptional target of MITF is the gene RRAGD, which encodes RagD, a protein implicated in the activation of the mTORC1 complex, a key amino acid-regulated metabolic hub that promotes protein synthesis and suppresses autophagy. By regulating RagD protein expression, MITF controls the recruitment of mTORC1 to lysosomes where it senses amino acids and consequently controls its activity. However, although regulation of RagD has been reported in Hela cells engineered to ectopically overexpress MITF (Di Malta et al., 2017), it has yet to be observed in a physiological setting in melanoma. Nevertheless, the regulation of RagD by MITF is a shared function with other MiT family members such as TFE3 and TFEB (Di Malta et al., 2017), but interestingly, it is not the only function that these related transcription factors share.

MITF, like TFEB, is a regulator of lysosomal biogenesis mediated by activation of AMPK, a kinase activated by high AMP:ATP levels. MITF can bind to the promoters of lysosomal and autophagosomal genes and plays a crucial role in the regulation of autophagy in melanoma cells in response to mitochondrial stress and starvation (Ploper et al., 2015; Fernández-Mosquera et al., 2017; Möller et al., 2019). Consistent with this, there is a positive correlation between the expression of MITF and lysosomal and autophagosomal genes in metastatic melanomas (Möller et al., 2019), suggesting that the activation of autophagy may be an adaptive response that increases melanoma cell survival upon nutritional stress.

Collectively, the current evidence strongly suggests that MITF works in melanoma as a central metabolic sensor that couples and integrates cell-extrinsic nutritional signals with cell-intrinsic metabolic responses to drive melanoma cells towards specific phenotypes that better guarantee melanoma survival under dynamic microenvironmental conditions.

In addition to its pro-proliferative role that is intimately related with its function as transcriptional regulator of a wide range of metabolic and cell cycle effectors, the MITF locus can be amplified up to 100-times in around 5%–20% of human melanomas (Garraway et al., 2005), supporting its function as lineage-addiction oncogene (Garraway et al., 2005). Of note, despite of such a dramatic gene amplification, the protein levels of MITF itself are only marginally increased, suggesting a limiting role of direct MITF gene amplifications in melanoma metabolic rewiring. However, it has been described that the long non-coding RNA SAMMSON is frequently co-amplified along with MITF locus (Leucci et al., 2016), but unlike MITF protein, SAMMSON RNA levels correlate with its gene copy number although its transcription is MITF-independent. Critically, SAMMSON RNA interacts with the protein p32, known to modulate mitochondrial metabolism in tumors, including melanoma (Fogal et al., 2008; Fogal et al., 2010). Depletion of SAMMSON decreases p32 mitochondrial location, leading to impaired mitochondrial membrane potential and generating abnormal mitochondria. Notably, the *in vivo* intravenous administration of antisense oligonucleotide targeting SAMMSON impairs tumor growth and enhances the pro-apoptotic effect of BRAF inhibitors. However, whether targeting SAMMSON will be also effective in metastatic melanoma requires further investigation.

Beyond MITF, other melanoma lineage-restricted factors, involved in coordinating metabolism and cell fate have been described. Indeed, the role of the neural crest lineage transcription factor SOX10 has recently emerged (Capparelli et al., 2022). SOX10 is, like MITF, heterogeneously expressed in melanoma (Rambow et al., 2018), and has been proposed as a regulator of phenotype switching in cutaneous melanoma (Capparelli et al., 2022) since its genetic ablation impairs proliferation and promotes the acquisition of invasive features and drug tolerance. Accordingly, melanoma cells lacking SOX10 display gene enrichment in EMT programs, as well as in metabolism and microenvironment-related pathways such as hypoxia and glycolysis (Capparelli et al., 2022). Of note, these effects on cell phenotype occur in parallel to the downregulation of MITF subsequent to SOX10 depletion. Whether they are connected to MITF or not, being fully or only partially dependent on SOX10 will require further investigation. Similarly, a possible direct involvement of SOX10 in regulating melanoma metabolic rewiring will also demand deeper attention.

## Flexibility in Nutrient Usage: Metabolic Rewiring Affecting Melanoma Progression

Nutrient uptake and processing are essential to life because they supply substrates for the production of energy and biomass. As tumor cells are proficient in proliferation and/or invasion, both energy-demanding processes, the role of nutrient availability in dictating cancer cell phenotypic states is a key issue in understanding cancer progression.

Nutrient availability in the TME is variable and dynamic and depends on several factors, such as lack of tumor vascularization, which creates regional nutrient deficiencies (Farnsworth et al., 2014); cell location within the solid tumor, which determines accessibility to irrigation and to nutrients from the extracellular space; or even cancer type and anatomical location where, for example, being close to fat depots can constitute an advantage for cancer cells to disseminate (Lengyel, 2010; Nieman et al., 2011; Lazar et al., 2016; Miranda et al., 2016; Tan et al., 2018; Zhang et al., 2018; Golan et al., 2019; Kersy et al., 2019). Therefore, the ability of cancer cells to adapt their metabolism according to nutrient availability in specific timeframes and locations will likely increase their chances of survival, highlighting that both nutrient access and metabolic flexibility are essential in determining cancer cell fate. As we will discuss, evidence suggests that metabolic rewiring is a hallmark of melanoma transformation and provides both the energetic support and capacity to build macromolecules that underpin phenotypic transitions essential for cancer cells to escape from a primary tumor, survive nutrient deficiency and eventually migrate and form metastases (Pavlova and Thompson, 2016; Rambow et al., 2018).

Proliferating cancer cells have increased demands for both glucose and glutamine, abundant nutrients in extracellular fluids that are key substrates for several metabolic pathways (Pavlova and Thompson, 2016). Although both metabolites can be *de novo* synthesized, very often cancer cells, due to their high nutritional requirements, depend on exogenous sources. Melanoma is not an exception, and a well-known metabolic feature of melanomas is the overexpression of the glucose transporter GLUT1, that has been related with both increased tumor growth and metastatic capacity (Koch et al., 2015).

A widely accepted view is that the metabolic advantage of cancer cells is mainly based on their ability to uptake exogenous glucose and to consume it as the main fuel through a glycolytic process uncoupled from mitochondrial oxidative metabolism, even in the presence of oxygen (Warburg and Negelein, 1923). Again, melanomas, even displaying very different oncogenic backgrounds, are not different from most of cancer cells, and in normoxic conditions they frequently show high glycolytic rates that increase further when they are subjected to a hypoxic environment (Ratnikov et al., 2016). Through this metabolic event, known as the “Warburg effect” or “aerobic glycolysis,” proliferative cells generate a large amount of glycolytic intermediates that will be used as primers of many other anabolic pathways (Heiden et al., 2009). As a consequence, when cancer cells need ATP, they may require a source of carbon different from glucose. Under these circumstances, glutamine can become a preferential metabolic substrate, as happens in melanomas refractory to chemotherapy (Hernandez-Davies et al., 2015; Baenke et al., 2016). Thus, glutamine addiction is a hallmark of melanoma (Filipp et al., 2012) that can be triggered by resistance to BRAF inhibitors (Hernandez-Davies et al., 2015; Baenke et al., 2016), and its consumption as an energetic substrate facilitates melanoma cell growth *via* energy-producing TCA cycle anaplerosis (Ratnikov et al., 2015). Moreover, glutamine can also provide additional building blocks required for proliferation, including glutamic acid, and is frequently depleted within melanomas leading to dedifferentiation (Pan et al., 2016; Falletta et al., 2017). Thus, increased uptake of glucose is just one of the many altered metabolic events taking place during cancer cell metabolic reprogramming (Yuneva et al., 2007; Fan et al., 2013).

Of relevance, both glucose and glutamine metabolism can lead to generation of the central carbon metabolite Acetyl Coenzyme A (Acetyl-CoA), essential for FA and cholesterol biosynthesis (Metallo et al., 2012; Mullen et al., 2012; Hosios et al., 2016). Indeed, proliferating cancer cells increase their lipid requirements to ensure membrane biosynthesis and rely on glucose and glutamine to obtain Acetyl-CoA and subsequently FAs. This is one of the reasons why in nutrient-rich environments, many proliferating tumors show increased FA biosynthesis and overexpression of lipogenic programs (Santos and Schulze, 2012; Currie et al., 2013; Gouw et al., 2019).

## Fatty Acid Metabolism as a Hallmark of Melanoma Aggressiveness

Despite the ability to enhance *de novo* generation of FAs from other available metabolites, increasing evidence shows that transformed cells have also an increased capacity to uptake FAs from external sources (Pascual et al., 2017; Zhang et al., 2018; Alicea et al., 2020). This double source of FAs, endogenous and exogenous, confers metabolic flexibility rather than redundancy, since thanks to this dual strategy tumors will be able to cover their high FA requirements even in environments where glucose, glutamine or both are limited. Moreover, the importance of FAs to cancer cell biology highlights, beyond the metabolic importance of glucose and glutamine, the significance of lipid metabolism as a potential key determinant of cancer cell fate. Thus, cancer cells endowed with metabolic plasticity that confers an ability to switch to FA metabolism have an adaptive advantage that will significantly favor their survival in microenvironments that have scarce, or are devoid of, glucose and glutamine.

As such, cells better able to cope with metabolically stressful environments represent good candidates to lead disease progression, including in melanoma. In fact, increasing evidence points to a central role of FA metabolism in tumor dissemination that goes beyond its classical involvement in cell proliferation. Indeed, FA metabolism is crucially implicated in invasion, dormancy, drug resistance ,and metastasis initiation (Nath and Chan, 2016; Pascual et al., 2017; Iwamoto et al., 2018; Zhang et al., 2018; Alicea et al., 2020; Vivas-García et al., 2020), all events that lead to poor prognosis in melanoma.

A study across more than 9,000 primary or metastatic tumor samples available in the TCGA database, including melanoma datasets, revealed the existence of a very strong association between FA metabolism gene signatures and EMT (Epithelial-to-Mesenchymal Transition) programs. A significant enrichment in genes involved in FA uptake, such as CAV1 or the FA transporter CD36, was found in metastatic tumors, revealing also a significant negative effect of FA uptake on patient survival rates (Nath and Chan, 2016). These data suggest that enhanced incorporation of exogenous lipids may be a strategy adopted by cancer cells, including melanoma, during metastatic dissemination.

Along the same lines, metastasis-initiating cells in melanoma and other cancer types show as a common feature the overexpression of CD36 (Pascual et al., 2017). Consequently, blockade of CD36 by genetic ablation or by using neutralizing antibodies reduces FA uptake in tumor cells and dramatically impairs metastasis formation and cancer aggressiveness in a wide variety of cancers including melanoma (Pascual et al., 2017; Ladanyi et al., 2018; Deng et al., 2019; Jiang et al., 2019; Pan et al., 2019; Pfeiler et al., 2019; Wang and Li, 2019; Watt et al., 2019). Supporting the relevance of this FA importer in invasive and metastatic melanoma *in vivo*, the invasive state induced by treatment with BRAF inhibitors observed by Rambow et al. (2018), which exhibits hallmarks of dedifferentiation and an invasive gene expression signature, exhibited high levels of CD36. This study also points to a possible contribution of CD36 in promoting drug tolerance, linking CD36 expression with tumor relapse.

Mechanistically, the presence of exogenous FAs derived from the diet or from adipocytes located in the proximity of the tumor can induce an increase in CD36 needed to promote EMT and metastasis initiation (Nath and Chan, 2016; Pascual et al., 2017; Ladanyi et al., 2018; Yang et al., 2018; Jiang et al., 2019; Pan et al., 2019), and interactions between FA uptake and *de-novo* FA synthesis might determine the efficacy of targeting CD36 to stop cancer progression (Watt et al., 2019). Moreover, a recent study proposes a new mechanism underlying the metastatic role of exogenous FA in oral carcinoma and melanoma by highlighting the unique role of the SFA palmitic acid (PA). PA-mediated CD36 overexpression induces epigenetic remodeling of melanoma cells that triggers the secretion of specific pro-neuroregenerative extracellular matrix from intratumoral Schwann cells, disruption of which blocks metastasis initiation (Pascual et al., 2021). Thus, PA-driven epigenetic changes in cancer cells boost metastatic progression by stimulating intratumor Schwann cells and innervation, features strongly associated with metastasis (Kuol et al., 2018; Zahalka and Frenette, 2020).

Beyond the role of PA as an epigenetic reprogramming driver, other FA like the MUFA Oleic Acid (OA), can increase the metastatic capacity of melanoma by decreasing oxidative stress and ferroptosis in cells migrating through the lymphatic system (Leong et al., 2011). Mechanistically, OA from lymph prevents ferroptosis in an Acyl-CoA synthetase long-chain family member 3 (ACSL3)-dependent manner (Ubellacker et al., 2020). ACSL3 activates FAs, preferentially OA, by transforming them into fatty acyl-CoA esters, which facilitates their incorporation into plasma membrane phospholipids (Tang et al., 2018) and dramatically reduces the sensitivity of plasma membranes to lipid peroxidation and subsequent ferroptosis (Magtanong et al., 2019). By conferring tolerance to ferroptosis in an ACLS3-dependent manner, OA increases the viability of melanoma cells migrating or seeding in lymphatic vessels or nodules, and therefore their ability to form more metastases compared to the melanoma cells circulating in the bloodstream (Ubellacker et al., 2020). Accordingly, higher ACSL3 expression in melanoma has been linked to poor prognosis in patients (Chen et al., 2016). However, contrary to expectations, CD36 overexpression was not detected in association with the protective role of OA in melanoma migrating cells (Ubellacker et al., 2020).

The current evidence therefore suggests that while FA internalization may be a metastatic trigger, it is not exclusively linked to CD36. In this regard, the participation of a different FA transporter, FATP1/SLC27A1, has also been shown to be crucial for melanoma progression and dissemination. Melanomas significantly overexpress this transporter on the tumor cell surface to uptake adipocyte-derived lipids, as its pharmacological inhibition impairs lipid internalization, thereby reducing tumor growth and invasion (Zhang et al., 2018). Furthermore, the involvement of different family members has been recently reported as relevant to age-related melanoma therapy resistance (Alicea et al., 2020). Melanoma cells can also uptake lipids from an aged TME through the induction of the FA transporter FATP2, generating specific age-related tolerance to BRAF/MEK inhibitors. Consequently, FATP2 blockade can overcome therapy tolerance and prevent tumor relapse (Alicea et al., 2020).

Regarding the usage of FA by melanoma cells, consequent to their increased uptake, it seems clear that the overexpression of FA transporters leads to a subsequent increase of mitochondrial FA Oxidation (FAO), and the coupling of both events promotes melanoma aggressiveness (Zhang et al., 2018; Alicea et al., 2020), suggesting that FA would constitute a central fuel for metastasis. Conversely, melanoma patients responding to immunotherapy show higher oxidative FA metabolism that leads to increased antigen presentation and therefore to better therapy efficacy that is blunted by knocking down mitochondrial FAO genes (Harel et al., 2019). These observations suggest that the activation of mitochondrial metabolism in general and FAO in particular, leads to increased immunotherapy sensitivity. However, further investigation is required to find new strategies that allow enhancement of FAO specifically in T cells, involved in the anti-tumor immune response (Waldman et al., 2020), without affecting melanoma cell lipid metabolism that could boost invasiveness and metastatic dissemination.

Importantly, FAs promote melanoma aggressiveness in ways beyond their impact on cellular energetics. FA incorporation into oxidative pathways is limited *in vitro* in metastatic melanoma cells that instead tend to use FAs to generate more complex structural and signaling lipids, such as ceramides (Louie et al., 2013) that are the predominant accumulated lipids in melanoma cells that develop therapy resistance (Alicea et al., 2020). Interestingly, ceramides are lipids that are often synthesized from an excess of PA and are frequently proposed as a trigger of cell migration and metastatic initiation. For example, non-invasive melanoma cells treated with either PA or Stearic Acid, another SFA, display a higher migratory capacity (Nomura et al., 2010; Pan et al., 2019).

An involvement of excess of SFA in cell de-differentiation, invasion and metastasis formation in melanoma has been also observed following treatment with specific inhibitors targeting the lipogenic enzyme SCD1, which uses SFAs as substrates to generate MUFAs. SCD1 inhibition therefore leads to accumulation of SFAs in melanoma cells which triggers an Integrated Stress Response (ISR) and the subsequent phosphorylation of one of its transducers, the eukaryotic translation Initiation Factor 2 alpha (p-eIF2α). This in turn induces a shut-down of global translation, but also promotes melanoma invasion and metastasis (Vivas-García et al., 2020). Importantly, metabolic stress such as glucose starvation (Ferguson et al., 2017), glutamine deprivation and inflammatory signals (Falletta et al., 2017) also converge on activation of the ISR and eIF2α phosphorylation to drive a melanoma switch from a proliferative to invasive phenotype. Activation of the ISR upregulates translation of the ISR effector Activating Transcription Factor 4 (ATF4) and represses MITF transcription and consequently reduces expression of its target SCD1 (Vivas-García et al., 2020) with both MITF and SCD also being translationally downregulated by eIF2 α phosphorylation (Falletta et al., 2017; Vivas-García et al., 2020). This suggests that an interplay between glucose, glutamine and FA metabolism is finely regulated in melanomas by nutrient and stress sensing pathways, such as the ISR that widely modulate basic cellular processes such as cap-independent translation to dictate cell fate.

## mTOR Pathway and its Interaction With the ISR in Melanoma

In a cancer cell, if on the one hand the ISR attenuates global mRNA translation to activate an alternative proteome to cope with oncogenic and microenvironmental stress, on the other hand activation of the mTOR pathway increases translation to match the proliferative demand. It is therefore plausible that the mammalian (or mechanistic) Target of Rapamycin mTOR pathway, a key sensor and effector of nutrient signaling as well as protein and lipid biosynthesis, plays an important role in melanoma progression, by itself or through a crosstalk with the ISR.

While we focus here on the role of mTOR regulation in melanoma, for a more comprehensive overview of the signaling pathways related to mTOR in physiological and pathological conditions we refer the reader to the recent reviews from Brunkard (2020), Liu and Sabatini (2020) and Popova and Jücker (2021).

mTOR is the catalytic subunit of two distinct multi-protein complexes, mTORC1 and mTORC2. mTORC1 is largely dedicated to amino acid sensing, it is inhibited by AMPK, the energy sensor of the cell, and it is activated by PI3K and MAPK signaling downstream from tyrosine kinase receptors (RTKs). mTORC2 also responds to PI3K and growth factor signaling and is activated by low glucose.

Mechanistically, in response to growth factors, nutrients, or oncogenic activation, mTORC1 activates protein synthesis on one side by phosphorylating the kinase (S6K) of ribosomal protein S6 (Hay and Sonenberg, 2004; Meyuhas, 2015), and on the other side by phosphorylating and inhibiting eIF4E-binding proteins (4E-BPs) which by competing with eIF2G for eIF4E binding disrupts eIF4F complex formation. Physiologically, mTOR-mediated phosphorylation of key components of the translation machinery regulates homeostasis through the increase of anabolic processes and decrease of catabolism in response to metabolic demands (Liu and Sabatini, 2020). 4E-BP phosphorylation confers protein synthesis rewiring which determines a moderate activation of global translation and a robust translation of specific mRNAs implicated in protein synthesis (Thoreen et al., 2012). Hyperactivation of the mTORC1 pathway has been observed in the majority of melanoma (Karbowniczek et al., 2008). Moreover, melanoma patients frequently display non-synonymous mutations of the MTOR gene, and such mutations can predict a worse prognosis (Yan et al., 2016). Related to this, recent evidence shows that activation of mTORC1 drives resistance to BRAF inhibitors in melanoma and suggest that BRAF/mTORC1 combinatorial therapy may improve patient survival by reducing the probability of relapse (Tran et al., 2021; Wang et al., 2021).

mTORC2’s primary role is through the regulation of AKT (Sarbassov et al., 2005) and members of the serum/glucocorticoid regulated kinase 1 (SGK) family (Jacinto et al., 2004), and is involved in cytoskeletal reorganization (Jacinto et al., 2004). A well characterized role of AKT is the ability to enhance glucose uptake (Ward and Thompson, 2012), and it has been recently demonstrated that low glucose activates an mTORC2-AKT axis (Li et al., 2018).

In melanoma, the PI3K pathway is activated through several mechanisms, both genetic and non-genetic, leading to mTORC1 activation (Tran et al., 2021). In BRAF mutated melanoma cell lines, the MEK/ERK pathway promotes the constitutive activation of mTOR through the p90 ribosomal S6 kinase RSK, inhibition of which abrogates tumor growth in mouse models (Romeo et al., 2013). The role of AMPK in inhibiting mTORC1 pathway has been confirmed in melanoma cell lines, whereby the AMPK activators AICAR and metformin can inhibit cell growth as well as anchorage-independent survival (Woodard and Platanias, 2010; Cerezo et al., 2013).

To complicate the picture, despite the apparent opposing effects of the ISR and mTORC1 signaling pathways, an intimate interconnection is emerging since 4E-BP1 is a transcriptional target of the ISR effector ATF4 (Yamaguchi et al., 2008; Kang et al., 2017). In the melanoma setting, BRAF inhibitors induce ATF4 translation through two independent signaling pathways: they trigger GCN2 kinase autophosphorylation that activates the canonical ISR (Nagasawa et al., 2017), but also can sustain mTOR- and eIF4B-driven ATF4 translation. The concerted action of these two pathways results in a cytoprotective effect (Nagasawa et al., 2020). Furthermore, mTORC1 signaling can activate ATF4, independently of the induction of the ISR, leading to purine synthesis (Ben-Sahra et al., 2016), glutathione synthesis and cystine uptake (Torrence et al., 2021).

Pathria and colleagues demonstrated that, in melanoma, adaptation to Lactate Dehydrogenase A LDHA inhibition, meant to disrupt the “Warburg effect,” is mainly associated with activation of the ISR, which in turn increases glutamine uptake and mTORC1 activation (Pathria et al., 2018).

A connection among the ISR and mTORC1 is reinforced by the work of Wengrod et al., 2015 who showed that mTORC1 inhibition leads to the GCN2-dependent activation of the ISR through the protein phosphatase 6 (PP6C), and that this activation is required for autophagy. Moreover, a subset of melanomas bearing PP6C mutations stabilize the wild type allele and by increasing the ISR, further stimulate autophagy *in vitro* and in human melanoma samples (Wengrod et al., 2015).

Beyond its traditional role as a master regulator of cancer anabolism, a new oncogenic role of mTORC1 has emerged as a regulator of invasiveness and metastatic dissemination by specifically enhancing the synthesis of proteins involved in migration. First elucidated in prostate cancer (Hsieh et al., 2012), this observation was thereafter confirmed in several other cancers, including melanoma. The impact of the mTORC1 signaling in driving melanoma cell migration has been recently demonstrated *in vitro* (Ciołczyk-Wierzbicka et al., 2020), whereby the use of the mTORC1 inhibitor, Everolimus, decreases metalloproteinase activity and invasion. Moreover, a specific mTORC2 inhibitor, JR-AB2-011, not only reduces proliferation and activates non-apoptotic cell death, but also impairs liver metastasis formation on a syngeneic murine metastasis model (Guenzle et al., 2021).

Despite the increasing evidence supporting a role of mTORC1 and mTORC2 in regulating the metastatic behavior of melanoma, further studies will be required to dissect the mechanisms by which these processes occur and to highlight their potential targetable vulnerabilities.

## Mitochondrial Metabolism in Melanoma

Tumorigenesis and metastatic dissemination rely on mitochondrial bioenergetics mainly through respiration, signaling and dynamics (Kumar et al., 2021). Mitochondria must sense the surrounding environment in order to adapt to cellular metabolic demands. Along the same lines, the cell must be aware of mitochondrial activity and comply with their needs through the activation of dedicated gene expression programs. Accordingly, systems for the bi-directional communication between mitochondria and the nucleus have evolved and anterograde signals and retrograde mitochondria-to-nucleus pathways act in concert to accommodate the specific metabolic requirements of a cancer cell (Shteinfer-Kuzmine et al., 2021).

Using a multi-omics analysis, Quiros and colleagues identified an ATF4-dependent retrograde signaling pathway which promotes mitochondria adaptation to stress by attenuating mitochondrial functions (Quirós et al., 2017). Further recent observations have clarified the importance of the ISR pathway in mitochondrial retrograde signaling, aimed at maintaining energetic homeostasis and mitochondrial integrity (reviewed by Kasai et al., 2019).

Since the mitochondrial genome encodes for only 13 proteins, which are core subunits of OXPHOS complexes, the bulk of mitochondrial proteins are encoded and transcribed in the nucleus, translated as precursors in the cytosol and then imported into the mitochondria (Bolender et al., 2008). Mitochondrial protein synthesis adapts to the influx of nuclear-encoded subunits (Richter-Dennerlein et al., 2016), therefore its proteome is entirely dependent on cytosolic translation and its regulation. Activation of the ISR pathway in melanoma cells promotes the selective translation of a subset of mRNAs encoding for mitochondrial proteins that generates an anterograde signaling from the cytosol to the mitochondria (Vendramin et al., 2021). Since therapy resistant cells show high levels of ISR activation, the authors suggest that this could explain the sensitivity of resistant cells to mitochondria-targeting agents such as uncouplers. They therefore propose the repurposing of antibiotics to inhibit mitochondria protein synthesis to target the resistant cells with a previous stratification of eligible patients according to the levels of ISR activation markers (Vendramin et al., 2021).

Drug resistance occurs in melanoma and can show either as unresponsiveness to therapy (e.g., only one third of patients benefit from immunotherapy) or as recurrence after targeted therapy (e.g., relapse after targeted therapy with Vemurafenib) (Tanda et al., 2020). While the mechanisms dictating unresponsiveness to both classical chemotherapy and immunotherapy are yet to be fully understood, it is very likely that post-therapy minimal residual disease (MRD) depends on both the genetic and phenotypic heterogeneity of melanoma (Pogrebniak and Curtis, 2018; Rambow et al., 2018). In this context, it is possible to reexamine the less recent literature to re-evaluate the many observations of altered mitochondrial metabolism and define its role in defining or conferring melanoma heterogeneity. For example, in 2013 it was discovered that following long-term *in vitro* exposure of melanoma cells to Vemurafenib or Cisplatin, a resistant slow-cycling pool of cells emerges, characterized by upregulation of OXPHOS compared to their sensitive counterparts (Roesch et al., 2013). Together with redirection of metabolites towards the TCA cycle, melanoma OXPHOS phenotype is increased by PGC1α. Haq et al. (2013) demonstrated an induction of mitochondrial biogenesis and OXPHOS upon small molecule-mediated inhibition of BRAF. This was dependent on the MITF/PGC1α axis which determines one of the adaptive metabolic programs leading to acquired resistance to targeted therapy. The data presented also offered a new therapeutic avenue as a potential hit in combinatorial targeting (Haq et al., 2013). Indeed, targeting mitochondrial biogenesis has been proposed as a strategy to overcome BRAFi resistance since *in vivo* studies show that a mitochondria-targeted HSP90 inhibitor is able to eradicate BRAFi resistant cells by inhibiting mitochondrial bioenergetics (Zhang et al., 2016). Furthermore, a close interaction between the Endoplasmic Reticulum (ER) and mitochondria has been observed in melanoma cells exposed to BRAFi, which facilitates calcium flux to mitochondria and attenuates ER stress-mediated cell death (Corazao-Rozas et al., 2016).

Melanoma cells bear the highest expression of PGC1α, compared to other cancer cell lines (Li et al., 2019), and high levels of PGC1α have a negative correlation with overall survival in melanoma patients (Vazquez et al., 2013). High levels of PGC1α expression in melanoma increase mitochondrial metabolism and are required for malignancy progression and survival (Vazquez et al., 2013; Luo et al., 2017). Surprisingly, respiration and mitochondria biogenesis induced by PGC1α are essential for dissemination and metastasis of several cancer cell lines, including melanoma (Lebleu et al., 2014). Moreover, increased OXPHOS has been identified in a zebrafish melanoma model of invasion and has been linked to the role of PGC1α in facilitating the switch from radial to vertical (invasive) growth (Salhi et al., 2020). These observations could lead to a hypothesis by which, following the acquisition of an invasive MITF-low phenotypic state leading to PGC1α inhibition and OXPHOS shut-down, melanoma cells face a metabolic emergency that may activate escape programs that induce cell migration towards nutrient enriched environments outside the tumor core.

On the other hand, melanomas expressing high PGC1α basal levels show increased metastatic capacity when PGC1α is suppressed (Luo et al., 2016). Therefore, although targeting mitochondrial oxidative metabolism has been initially considered a good therapeutic strategy to prevent melanoma proliferation (Lim et al., 2014; Luo et al., 2017), on the contrary targeting PGC1α activity may be detrimental for disease progression as low levels of PGC1α can drive melanoma invasiveness and metastasis (Luo et al., 2016).

It is possible that the double role of PGC1α in promoting tumor growth and suppressing metastasis is dependent on the phenotypic state of the melanoma cell, and therefore its metabolic requirements. In the proliferative MITF-high state PGC1α favors oxidative metabolism, a proficient and economic way of supplying energy. In the invasive MITF-low state PGC1α depletion rewires melanoma cells towards metabolic dependency on HIF1α-mediated glycolysis and glutamine usage (Lim et al., 2014). Finally, since growth of the metastatic lesion requires the high energy-demanding process of proliferation reactivation, MITF re-upregulation and consequent upregulation of PGC1α-driven OXPHOS are required (Beleaua et al., 2021). Collectively, these findings suggest that there might be a narrow range of variation for PGC1α levels to confer an energetic advantage to cells to either promote proliferation or metastatic progression.

Remarkably, PGC1α also exerts a ROS scavenging effect through the induction of many ROS-detoxifying enzymes, such as SOD2 and GPX1 (St-Pierre et al., 2006). Indeed, Vazquez et al. (2013), identified a subset of melanoma with high levels of PGC1α expression, and demonstrated that they able to tolerate oxidative stress better than their PGC1α low counterpart. A fundamental ROS detoxifying role played by the lactate transporter MCT1 has been recently outlined in melanoma, whereby high levels of MCT1 identify a subset of efficiently metastasizing cells capable to uptake circulating lactate, direct it to the oxidative pentose phosphate pathway relative to glycolysis and decrease oxidative stress (Tasdogan et al., 2020).

On the other hand, it appears that oxidative stress could promote cell migration and invasion in lung cell carcinoma (Ishikawa et al., 2008) as well as in melanoma through superoxide-dependent activation of SRC and PYK2 (Porporato et al., 2014). Oxidative stress tolerance is of vital importance for metastasizing melanoma cells. Indeed, patient-derived melanoma cells tested in an immunodeficient mouse model experience high levels of oxidative stress in the blood and visceral organs compared to their subcutaneous counterparts (Piskounova et al., 2015). Therefore, metabolic changes are required to withstand oxidative stress during metastatic dissemination, such as activation of the folate pathway to increase NADPH generation (Piskounova et al., 2015). Unbalanced mitochondrial metabolism could therefore have the double role of promoting metastasis through ROS production and increasing survival by detoxifying them once a melanoma cell is circulating in the bloodstream or has reached the seeding organ. These observations suggest the important role of the reversibility and adaptability of metabolic changes to accommodate the melanoma needs depending on the microenvironment.

Since ROS are drivers of metabolic stress, a role of the ISR in regulating ROS detoxification has been elucidated, whereby ATF4 is capable of activating an antioxidant response through transcription of the major antioxidant enzymes HO-1 (Dey et al., 2015) and glutathione (Torrence et al., 2021). Enhanced ROS detoxification is not only a key step in the metastatic process, but it is an important mediator of tumorigenesis related to oncogene activation. Indeed, activating mutations on BRAF and KRAS oncogenes suppress oxidative stress through the NRF2 antioxidant program (Denicola et al., 2011), while HER2 overexpression induces generation of antioxidants through the activation of the pentose phosphate pathway (Schafer et al., 2009), facilitating survival and proliferation of cancer cells.

Finally, a role of mitochondria dynamics is emerging in regulating melanoma tumorigenesis and metastasis. As an example, markers of mitochondria fission and fusion such as dynamin-related protein 1 (DRP1), mitochondrial fission protein 1 (FIS1), and mitofusins are upregulated in patient tumor samples compared to their healthy counterparts and are significantly associated with metastasis (Soares et al., 2019). Moreover, levels of TMX1 and TMX3, transmembrane proteins with oxidoreductase activity which promote ER-mitochondria communication, are upregulated in melanoma cells and patient samples and are associated with poor disease outcome (Zhang et al., 2019).

## Melanoma Metabolism, a Tool to Boost Immunotherapy

A major driver of therapy resistance is phenotypic heterogeneity arising as a consequence of adaptation to microenvironmental cues, including nutrient limitation, inflammation, and crucial in the clinical setting, exposure to chemotherapy itself (Rambow et al., 2019). Facing this landscape, immunotherapy has emerged as a cancer treatment able to achieve a significant increase in patient survival rates, especially for cancers like melanoma that display a high tumor mutational load (Alexandrov et al., 2013; Yang, 2015). Although promising, a significant proportion of patients do not respond to immunotherapy (Hölzel et al., 2013; Zikich et al., 2016). Understanding the mechanisms by which T cell activation and the consequent cancer cell clearance can be achieved is therefore of a special interest, and a role for metabolic adaptation is emerging. Since an association between tumor metabolic state and the tumor response to immunotherapy may exist (Buck et al., 2017; Cascone et al., 2018; Harel et al., 2019; Indini et al., 2021), targeting lipid metabolism may be a way to increase sensitivity to immunotherapy. For example, a recently performed proteomic analysis of 116 advanced melanomas from patients treated with immunotherapy [tumor infiltrating lymphocyte-(TIL) or anti-PD1 therapies] has shown that the response to immunotherapy is associated with enriched mitochondrial lipid metabolism (Harel et al., 2019). This result strongly supports the idea that elevated mitochondrial FAO leads to increased antigen presentation and IFN signaling to facilitate T cell-mediated cancer cell death. Moreover, increased mitochondrial metabolism and FAO in T cells, improves T cell survival and functionality, enhancing sensitivity to anti-PD-1 therapy (Chowdhury et al., 2018; Zhang et al., 2021) in other cancer types. Interestingly, a similar induction of IFN signaling has been revealed using RNA-seq analysis in human melanoma cells and mouse B16 melanoma treated with specific inhibitors of the FA desaturase SCD1. Thus, SCDi triggered an ISR and NF-κB activation that generated a pro-inflammatory environment (Vivas-García et al., 2020) able to attract immune cells that would further increase inflammation within the tumor. This suggests that targeting FA metabolism may be an approach to increase cancer cell sensitivity to immunotherapy, especially in the so-called cold immune cell-poor tumors, turning them into hot tumors that are responsive to T-cell therapy. However, a limiting aspect may be that the ISR, acutely triggered in melanoma by glucose or glutamine limitation (Falletta et al., 2017; Ferguson et al., 2017), SCD1 inhibition (Vivas-García et al., 2020) and inflammation (Falletta et al., 2017), induces PD-L1 translation and suppresses anti-tumor immunity (Suresh et al., 2020). Moreover, such a mechanism may explain why melanomas enriched in immune cells that normally express low levels of MITF may not have an efficient anti-tumor immune response (Ballotti et al., 2020).

Further research is therefore necessary to determine whether using therapies targeting lipid metabolism in combination with immunotherapy may be effective by promoting the desirable responses while avoiding the invasive and metastatic effects that are also associated with, for example, long-term SCD inhibition or stimulation of FAO.

## Concluding Remarks

**FIGURE 2:**
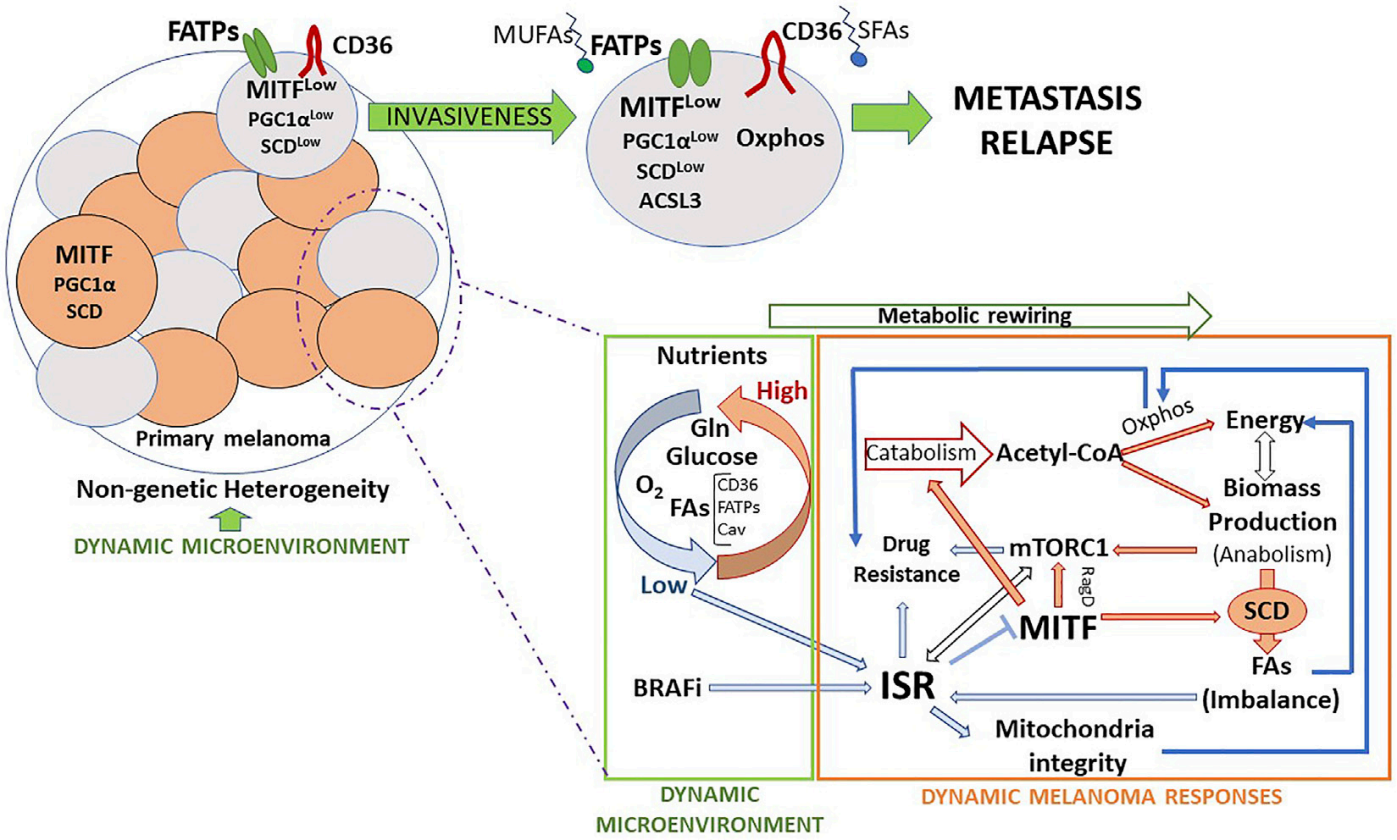
Metabolic features and routes towards invasion and metastasis. Melanomas, subjected to microenvironment-driven non-genetic heterogeneity, are composed of several cell subsets, which express different levels of MITF and display specific metabolic features that provide a distinct identity. Eventually, as a consequence of such a reversible identity and as a first step of the metastatic process, some cells acquire invasive capacity and escape from the primary tumor. Migrating cells can adapt to the extrinsic insults affecting them. FA metabolism may provide melanoma cells with survival advantages that facilitate the metastatic process. The main dynamic microenvironmental cues that lead to melanoma phenotypic changes are nutrients, drugs, and inflammatory signals (not represented in the figure). Melanoma cells respond to changes in these parameters adapting their metabolism, at least in part, through the activation of the ISR. The ISR works in melanoma as a microenvironment-sensing pathway that modulates cell phenotype and metabolic activity, alone or in coordination with other key nutrient sensing pathways, like mTORC1.

Despite being a highly inefficient process, metastasis is the main cause of cancer death for a wide variety of cancer types. Melanoma represents a paradigm for understanding disease progression including metastatic dissemination, since despite its low frequency there are excellent markers that highlight specific phenotypic subpopulations. In this review we have described many of the recently identified mechanisms of metastatic dissemination which rely on metabolic rewiring and the main pathways regulated by and regulating it (Figure 2). We shed light on the complexity and the interconnection between the metabolic processes and how they govern the phenotypic changes in the melanoma cells. Moreover, we highlight how the microenvironment and the metabolic plasticity of the cells that populate it play a crucial role in conferring the invasive potential and survival advantage necessary for a melanoma cell to disseminate and colonize a distant organ. Finally, we want to conclude by remarking that phenotypic heterogeneity, one of the major challenges for therapy efficacy, means metabolic plasticity, and adaptation. This notion highlights the role of cross-talk between oncogenic drivers and metabolic pathways which can offer innovative insights into melanoma therapeutic opportunities. With this overview we want to highlight the necessity for a deeper understanding of the mechanisms governed by the metabolic changes in search for new targetable vulnerabilities that improve the efficacy of the current therapeutic strategies. Indeed, clinical intervention on tumour metabolism is a promising and challenging approach, and further advances in metabolic evaluation of melanoma patient samples with metabolic imaging and quantification could bring forward the development of diagnostic and prognostic markers and specific therapeutic strategies that would be potentially more effective in the eradication of this disease.

However, specifically targeting the metabolic vulnerabilities of cancer cells without affecting the healthy population is a major challenge that needs to be addressed. Furthermore, phenotypic heterogeneity poses a challenge also for targeting metabolic pathways. Indeed, the coexistence of metabolically different populations within the same tumour may hamper the complete eradication of the tumour mass when using targeted therapies. The balance between the targetable metabolic pathways and their specificity could provide a new avenue of treatment. Another potentially valuable approach could derive from pharmacologically directed phenotype switching to drive melanoma cells toward a unique specific and targetable metabolic phenotype (Sáez-Ayala et al., 2013). While this approach involving perturbation of the dynamic metabolic equilibrium of melanoma cells may offer interesting therapeutic opportunities, extreme care is needed in considering the efficacy of this therapeutic strategy. As an example, chemically increasing the ISR pathway could result in more cell death but on the other hand could trigger the metastatic potential of the cells that might enhance disease progression (Licari et al., 2021).
